# *XAP5 CIRCADIAN TIMEKEEPER* Positively Regulates *RESISTANCE TO POWDERY MILDEW8.1*–Mediated Immunity in *Arabidopsis*

**DOI:** 10.3389/fpls.2017.02044

**Published:** 2017-11-30

**Authors:** Yong-Ju Xu, Yang Lei, Ran Li, Ling-Li Zhang, Zhi-Xue Zhao, Jing-Hao Zhao, Jing Fan, Yan Li, Hui Yang, Jing Shang, Shunyuan Xiao, Wen-Ming Wang

**Affiliations:** ^1^Rice Research Institute and Research Center for Major Crop Diseases, Sichuan Agricultural University, Chengdu, China; ^2^Institute for Bioscience and Biotechnology Research and Department of Plant Sciences and Landscape Architecture, University of Maryland, College Park, College Park, MD, United States; ^3^Collaborative Innovation Center for Hybrid Rice in Yangtze River Basin, Sichuan Agricultural University, Chengdu, China

**Keywords:** *RPW8.1*, *XCT*, powdery mildew, cell death, disease resistance

## Abstract

Ectopic expression of the Arabidopsis *RESISTANCE TO POWDERY MILDEW8.1* (*RPW8.1*) boosts pattern-triggered immunity leading to enhanced resistance to different pathogens in Arabidopsis and rice. However, the underlying regulatory mechanism remains largely elusive. Here, we report that *XAP5 CIRCADIAN TIMEKEEPER* (*XCT, At2g21150*) positively regulates *RPW8.1*-mediated cell death and disease resistance. Forward genetic screen identified the *b3-17* mutant that exhibited less cell death and susceptibility to powdery mildew and bacterial pathogens. Map-based cloning identified a G-to-A point mutation at the 3′ splice site of the 8th intron, which resulted in splice shift to 8-bp down-stream of the original splice site of *XCT* in *b3-17*, and introduced into a stop codon after two codons leading to a truncated XCT. *XCT* has previously been identified as a circadian clock gene required for small RNA biogenesis and acting down-stream of *ETHYLENE-INSENSITIVE3* (*EIN3*) in the ethylene-signaling pathway. Here we further showed that mutation or down-regulation of *XCT* by artificial microRNA reduced *RPW8.1*-mediated immunity in R1Y4, a transgenic line expressing *RPW8.1-YFP* from the *RPW8.1* native promoter. On the contrary, overexpression of *XCT* in R1Y4 background enhanced *RPW8.1*-mediated cell death, H_2_O_2_ production and resistance against powdery mildew. Consistently, the expression of *RPW8.1* was down- and up-regulated in *xct* mutant and *XCT* overexpression lines, respectively. Taken together, these results indicate that *XCT* positively regulates *RPW8.1*-mediated cell death and disease resistance, and provide new insight into the regulatory mechanism of *RPW8.1*-mediated immunity.

## Introduction

To prevent the invasion of pathogenic microbes, plants have evolved two major defense systems in addition to pre-formed barriers such as cell walls and leaf hairs (Jones and Dangl, 2006). The first system is termed pathogen-associated molecular pattern (PAMP)-triggered immunity (PTI), which is activated when the receptors on the surface of host cells perceive conserved PAMPs (Zipfel et al., 2006; Boller and Felix, 2009). PTI is featured by a series of defense responses, including burst of reactive oxygen species (ROS), deposition of callose, induction of defense–related genes (Ebel and Mithöfer, 1998; Asai et al., 2002; Zipfel et al., 2006). However, adapted pathogens can subvert PTI by using virulent effectors (Dou and Zhou, 2012). In turn, plants employ the second defense system, called effector-triggered immunity (ETI) that is activated upon recognition of pathogen effectors by plant intracellular nucleotide-binding and leucine-rich repeat receptors (NLRs) known as resistance (R) proteins (Spoel and Dong, 2012; Dangl et al., 2013). Defense responses in ETI are stronger than those in PTI and often culminate in hypersensitive response (HR) at the infection site (Greenberg and Yao, 2004; Dangl et al., 2013).

Most identified plant *R* genes encode proteins possessing an intracellular nucleotide-binding site and leucine-rich repeat (NBS-LRR) domain (Bonardi et al., 2012) or an extracellular LRR (eLRR) domain (Dangl and Jones, 2001). The R proteins can activate race-specific resistance via direct or indirect interaction with their cognate effectors (Dodds et al., 2006; Krasileva et al., 2010). A few *R* genes encode atypical R proteins which are structurally different from the typical R proteins (NBS-LRRs and eLRRs), and mediate broad-spectrum and/or durable resistance to single or multiple pathogens. For example, tomato *Pto* encodes a serine-threonine protein kinase conferring resistance to *Pseudomonas syringae* pv. tomato (Martin et al., 1993). Wheat *Lr34* encodes a putative ABC transporter protein conferring resistance to wheat rust and powdery mildew (Krattinger et al., 2009). Barley *Rpg1* encodes a receptor kinase-like protein with two tandem kinase domains conferring resistance to barley stem rust fungus (Brueggeman et al., 2002).

The Arabidopsis *RESISTANCE TO POWDERY MILDE RPW8.1* (*RPW8.1*) and *RPW8.2* encode non-NLR R protein with a putative trans-membrane or signal peptide domain and one or two coiled-coil motifs (Xiao et al., 2001; Wang et al., 2013). *RPW8.1* and *RPW8.2* share 45% identity and 65% similarity in amino acid sequences and confer broad-spectrum resistance against all tested infectious powdery mildew isolates in Arabidopsis (Xiao et al., 2001). While RPW8.2 is specifically targeted to the extra-haustorial membrane encasing the haustorium of powdery mildew in the invaded epidermal cells, RPW8.1 is found in a membranous structure peripheral to the chloroplasts in the mesophyll cells (Wang et al., 2007, 2009). In addition, the transgenic Arabidopsis plants expressing RPW8.1-YFP exhibit discretely spontaneous cell death-caused pits on the adaxial side of leaves and display enhanced resistance to virulent powdery mildew, oomycete and bacterial pathogens (Ma et al., 2014; Li et al., 2017). Although the full function of *RPW8.1* relies on the properly expressed ASYMMETRIC LEAVES1 (AS1), a MYB domain transcription factor functioning in regulation of leaf cell fate (Zhao et al., 2015), the regulatory mechanism of *RPW8.1*-mediated immunity remains largely unknown.

In Arabidopsis, *X-CHROMOSOME ASSOCIATED PROTEIN5* (*XAP5*) *CIRCADIAN TIMEKEEPER* (*XCT*) is a single copy gene encoding a nuclear protein highly conserved across eukaryotes. *XCT* functions in regulation of circadian rhythms, ethylene responses and small RNA production (Martin-Tryon and Harmer, 2008; Ellison et al., 2011; Fang et al., 2015). Loss-of-function mutations in *XCT* lead to short-period circadian rhythms, delayed greening, and altered regulation of ethylene responses in the aerial tissues (Martin-Tryon and Harmer, 2008; Ellison et al., 2011). *XCT* also regulates production of small RNAs via modulating the expression of *DCL1, DCL3* and *DCL4* (Fang et al., 2015). Because small RNAs, circadian rhythms, and ethylene-signaling pathway play roles in plant innate immunity, it is anticipated that *XCT* may be involved in defense. However, robust evidence is currently lacking in the literatures.

To identify components involved in regulation of *RPW8.1*-mediated immunity, we performed a forward genetic screen for mutants with either enhanced or compromised cell-death phenotypes using ethyl methane sulfonate (EMS)-mediated mutagenesis based on the transgenic line R1Y4 that expressed RPW8.1-YFP from its native promoter. Previously, we reported that proper expression of *AS1* is required for *RPW8.1*-mediated defense against powdery mildew (Zhao et al., 2015). Here, we described the isolation of the *b3-17* mutant that exhibited a smaller plant stature and compromised resistance to powdery mildew. By map-based cloning, we found that the *b3-17* mutant contains a novel allele of *XCT* (*At2g21150*) in R1Y4. Then we made transgenic lines with reduced expression or increased expression of *XCT*. Our data demonstrated that down-regulation of *XCT* resulted in reduced *RPW8.1*-mediated immune responses, whereas, overexpression of *XCT* led to enhanced *RPW8.1*-mediated immunity. Therefore, *XCT* acts as a positive regulator in the *RPW8.1*-mediated defense pathway.

## Materials and Methods

### Plant Materials and Growth Conditions

The transgenic Col-*gl* (Col-0 containing the glabrous mutation) line R1Y4 expressing RPW8.1-YFP from Ma et al. (2014) was used for EMS mutagenesis (Zhao et al., 2015). The *b3-17* mutant was isolated for its dwarf phenotype. The *xct-5* mutant was derived from crossing *b3-17* with Col*-gl.* All seeds were sowed on 1/2 (W/V) Murashige Skoog (MS) basal media containing appropriate antibiotics and treated at 4°C for 2 days. Seedlings were transplanted into peat soil (Pindstrup, Beijing) after appearance of true leaves and were put in a growth chamber under the conditions of 23°C, 75% relative humidity, short-day (10 h light, 14 h dark) for vegetative growth or long-day (14 h light, 10 h dark) for induction of flowering or after inoculation of powdery mildew.

### Map-Based Cloning of *b3-17*

In order to generate enough polymorphism for linkage analysis of the mutated locus in *b3-17* that was in Col-*gl* background, we constructed an F_2_ segregating population by crossing *b3-17* with Landsberg erecta (Ler). Then the individuals displaying mutant phenotype were selected for gene mapping. The simple sequence length polymorphism (SSLP) markers and new SNP markers (Supplementary Table S1) were used for initial mapping. After the mutation was mapped between the markers F26H11 and F7O24, the candidate genes were sequenced leading to the identification of a G-to-A mutation at the 3′ splice site of the 8th intron of *XCT* (*At2g21150*) (**Supplementary Figure S1**).

For complementation test, we amplified the wild type *XCT* containing the 2090-bp sequences upstream of the start codon and the 522-bp sequences downstream of the stop codon with the primers EcoRXCT-F and KpnXCT-R. We chose this region as the wild type *XCT* because the upstream sequences contain a predicted transcriptional start site and may function as the native promoter of *XCT* as reported in a previous paper (Martin-Tryon and Harmer, 2008) and the downstream sequences contain the 3′-untranslated region (UTR). The amplified fragment was cloned into the binary vector pCAMBIA1300 at the *Eco*RI*/Kpn*I site generating the plasmid pP_XCT_:XCT. The plasmid was introduced into *b3-17* and *xct-5* via Agrobacterium-mediated floral dip according to a previous approach (Clough and Bent, 1998). Positive transformants were screened on 1/2 (W/V) MS basal media containing 35 mg/L of Hygromycin B (Roche, United States).

### Construction of Transgenic Lines Down- and Over-Expressing *XCT*

To make transgenic lines with reduced expression of *XCT* through artificial microRNA (amiRNA), *XCT* specific primers were designed with the WMD Web MicroRNA Designer^1^. The construct expressing *amiRXCT* targeting the 3′-UTR of *XCT* was made according to Schwab et al. (2006) using template plasmid pRS300 and primers XCTmiR-sI, XCTmiR-aII, XCTmiR^∗^sIII, and XCTmiR^∗^aIV (Supplementary Table S1) (Schwab et al., 2006). The resulting amiRNA was cloned into pKANNIBAL-35S-RBS (courtesy of Yuelin Zhang) at *Eco*RI /*Bam*HI site generating the plasmid pamiRXCT. To make transgenic lines over-expressing *XCT*, the coding sequences of *XCT* was amplified with the primers Kpn-oeXCT-F and Kpn-oeXCT-R (Supplementary Table S1), and cloned into the binary vector pCAMBIA1300-35S at *Kpn*I site leading to the plasmid p35S::XCT. The constructs were transformed into the agrobacterium strain GV3101 together with the helper plasmid pSOUP and then introduced into R1Y4 and Col-*gl*, respectively, via Agro-mediated floral dip (Clough and Bent, 1998). Positive transformants were screened on 1/2 MS media containing 35 mg/L of Hygromycin B, and the expression level of *XCT* was tested by quantitative reverse transcription PCR (qRT-PCR).

### RNA Extraction and qRT-PCR Assay

Total RNA was extracted from 80 mg leaves with TRIzol^®^ Reagent (Invitrogen). For time course analysis, leaves were collected at 0, 3, 6, 12, 24 h after treatment with 2 μM of flg22. For qRT-PCR assay, 500 ng total RNA was used for cDNA synthesis using ReverTra Ace^®^ qPCR RT Master Mix with gDNA Remover (Toyobo). Quantitative RT-PCR was performed with gene-specific primers (Supplementary Table S1) and QuantiNova^TM^ SYBR^®^ Green PCR Reagent (Sigma) in Bio-Rad CFX96^TM^ Real-Time system. *ACT2* was used as an internal reference. Quantification of fold change was calculated by the 2[-Delta Delta C(T)] Method (Livak and Schmittgen, 2001). Statistical analysis was performed by *t*-test or by a one-way ANOVA followed by *post hoc* Tukey HSD analysis. Quantitative data were processed using Sigma Plot 10.0.

### Pathogen Inoculation and Microscopy Analyses

Powdery mildew isolate *Golovinomyces cichoracearum* UCSC1 was maintained on live *pad4-1 sid2-1* plants. Inoculation, visual scoring, and quantification of disease susceptibility were done as described previously (Xiao et al., 2003). For bacterial proliferation assay, leaves of 5-week-old plants were infiltrated with the virulent strain *P. syringae* DC3000 (OD_600_ = 0.0005), and the non-pathogenic strain *P. syringae* DC3000(*hrcC^-^*) (OD_600_ = 0.002) according to a previous report (Li et al., 2017). Bacterial growth was determined by colony counting as previously described (Zipfel et al., 2004).

Dying cells in inoculated leaves were analyzed by trypan blue (TB) staining at 10 days post inoculation (dpi) of powdery mildew using a method modified from Xiao et al. (2003). Briefly, inoculated leaves were soaked in TB solution, and boiled for 2 min, then de-stained using Chloral Hydrate (Sigma). For examination of H_2_O_2_ production by 3,3′-diaminobenzidine (DAB) staining, inoculated leaves were cut at petiole, submerged in 1 mg /ml of acidic DAB solution (Sigma), vacuum-infiltrated for 3 min at 0.2 gk/cm^2^, incubated overnight in dark, and destained using cleaning solution. Images were acquired under Canon EOS Rebel T2i and Zeiss Imager A2.

For microscopy of the accumulation of RPW8.1-YFP, leaves from 5-week-old plants were examined under a laser scanning confocal microscope (Nikon A1) according to a previous report (Huang et al., 2014). All confocal graphs presented in the manuscript were two-dimensional projections of 10–30 Z-stack images. Images were processed using Image J, NIS Elements C and/or Adobe Photoshop CS5.

### Protein Extraction and Western Blotting Analysis

Total protein was extracted from 200 mg fresh leaves in extraction buffer supplemented with 100 mM PMSF, 1% (V/V) Triton X-100 following a previous report (Wang et al., 2012). After separation by 10% SDS-PAGE, the denatured protein was transferred to Immobilon^®^-P Transfer Membrane (Millipore), and subjected to blot analysis using polyclonal anti-sera of GFP (1:2000) (BBI) and ClarityTM Western ECL Substrate system (BIO *RAD*) to detect RPW8.1-YFP.

## Results

### The *b3-17* Mutant Contains a Novel *XCT* Allele

**FIGURE 1:**
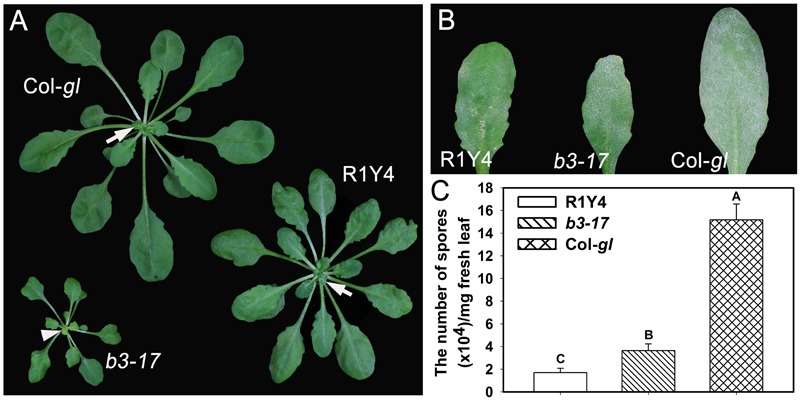
The *b3-17* mutant impairs *RPW8.1*-mediated defense against powdery mildew. **(A)** Representative 6-week-old plants of the indicated lines show the difference in plant size. Note the freshly emerged light-green leaf (arrowhead) in the *b3-17* mutant in comparison with the green ones (arrows) in R1Y4 and Col-*gl*. **(B)** Representative leaves from the indicated lines show the disease phenotype of powdery mildew at 10 days post inoculation (dpi). **(C)** Quantitative assay for the powdery mildew sporulation at 10 dpi. The number of spores was counted from 30 mg infected leaves for statistical analysis. Error bars represent standard deviation (SD, *n* = 6). Different letters above the bars indicate significant differences at *P* < 0.01. Similar results were obtained in three independent experiments.

Previously, we found that ectopic expression of *RPW8.1* leads to enhanced resistance against different pathogens via boosting pattern-triggered immunity (Ma et al., 2014; Li et al., 2017). In order to identify regulators for *RPW8.1*-mediated immunity, we performed a forward genetic screen and isolated the *b3-17* mutant that exhibited compromised cell death and smaller plant stature in comparison with R1Y4 (**Figure 1A**). The *b3-17* mutant also exhibited susceptibility to powdery mildew as indicated by the white fungal mass on the leaf surface (**Figure 1B**). Quantification of spores at 10 dpi confirmed the susceptibility of *b3-17* to powdery mildew, because the sporulation on *b3-17* was significantly higher than that on R1Y4, although lower than that on Col-*gl* (**Figure 1C**). These results demonstrated the impairment of *RPW8.1*-mediated resistance against powdery mildew in the *b3-17* mutant.

**FIGURE 2:**
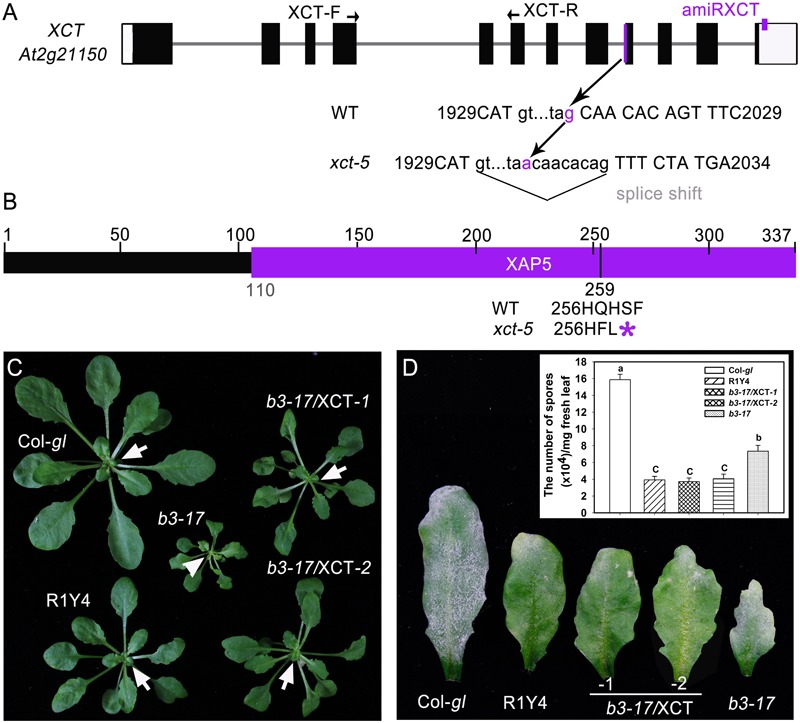
The *b3-17* mutant contains a novel allele of *XCT.* **(A)** Schematic gene structure of *XCT* (*At2g21150*) and the position of the mutation site in *b3-17*. XCT-F and XCT-R indicate the position of primers for quantitative RT-PCR (qRT-PCR). A small purple box indicates the target site of artificial microRNA at the 3′-untranslated region. **(B)** Predicted protein structure of XCT and the stop codon introduced by the mutation in *b3-17* was indicated (^∗^). The purple bar represents the X-chromosome Associated Protein 5 (XAP5) domain. **(C,D)** Representative plants **(C)** and leaves **(D)** of the indicated lines show the phenotypic complementation before **(C)** and after **(D)** inoculation of powdery mildew. *b3-17*/XCT indicates the complemented lines by introduction of the wild type *XCT* gene into the *b3-17* mutant. Note the freshly emerged light-green leaf (arrowhead) in the *b3-17* mutant in comparison with the green ones (arrows) in the other lines. Inset in **(D)** shows quantification analysis on sporulation of powdery mildew from the indicated lines. Error bars indicate SD (*n* = 3). Different letters above the bars indicate significant differences at *P* < 0.01. Similar results were obtained in two independent experiments.

The *b3-17* mutation was initially mapped to the chromosome 2 between the two simple sequence length polymorphism markers F6F22 and F2G1 (**Supplementary Figure S1A**). By using additional markers, the mutation was mapped to a 10.7-kb region between the markers F26H11 and F7O24. Finally, sequencing candidate genes in this region identified a G-to-A mutation at nucleotide 2016 of *XCT* (*At2g21150*) in *b3-17*, which occurred at the 3′ splice site of intron 8 (**Figure 2A** and **Supplementary Figure S1B**). Further sequencing *XCT* cDNA from *b3-17* revealed that this mutation resulted in a splice shift of eight nucleotides downstream of the original splice junction (**Figure 2A** and **Supplementary Figure S1C**), generating a mRNA encoding a truncated XCT lacking the C-terminal 79 amino acid residues that covers one-third of the conserved X-chromosome Associated Protein 5 (XAP5) domain (**Figure 2B** and **Supplementary Figure S1D**).

To confirm that the *b3-17* mutant phenotypes were due to the point mutation in *XCT*, we introduced the wild type *XCT* gene containing the 2090-bp sequences upstream of the start codon and 522-bp sequences downstream of the stop codon into *b3-17*. This wild type *XCT* includes the native promoter and 3′-UTR (Martin-Tryon and Harmer, 2008). The obtained >20 transgenic plants were all restored to the phenotypes of R1Y4, including plant size and defense against powdery mildew (**Figures 2C,D**). These data indicate that the *b3-17* contains a novel allele of *XCT*. Because there are four *xct* mutant alleles reported previously (Martin-Tryon and Harmer, 2008; Fang et al., 2015), we designated this allele *xct-5* and renamed *b3-17* as R1Y4/*xct-5*.

### Mutation or Down-Regulation of *XCT* Compromises *RPW8.1*-Mediated Defense Responses

**FIGURE 3:**
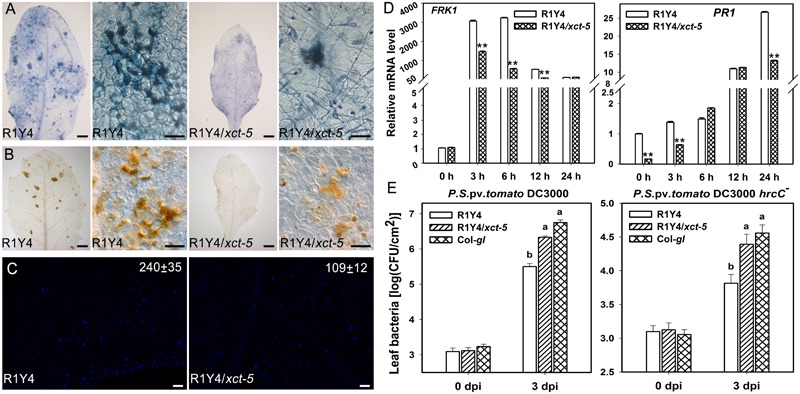
Mutation in *XCT* compromises *RPW8.1*-mediated immunity. **(A,B)** Representative infected leaves and leaf sections show fungus-induced cell death **(A)** and H_2_O_2_ production **(B)** in the indicated lines at 8 days post inoculation (dpi) of powdery mildew. Scale bars, 50 μm. **(C)** Flg22-induced callose deposition in the indicated lines. Leaves from 5-week-old plants were injected with 2 μM of flg22 and samples were stained with 0.01% aniline blue at 12 h post injection. Scale bars, 10 μm. Image J was used for quantification of callose deposition. The average number of callose deposits (±SE) from four different leaves is indicated in the images. Similar pictures were captured from more than three infected lines and similar results were obtained in two independent experiments **(A–C)**. **(D)** Quantitative RT-PCR analysis on the expression of the indicated defense-related marker genes in R1Y4/*xct-5* and R1Y4 at 0, 3, 6, 12, 24 h after treated by flg22. Transcript levels were normalized to that in R1Y4 at 0 h. Error bars indicate SD (*n* = 3). Asterisks ^∗∗^ above the bars indicate significant difference between *b3-17* and R1Y4 (*t*-test, *P* < 0.01). **(E)** Bacterial growth assay on the indicated plants. Fully expanded leaves of the indicated plants were injected with the virulent strain *Pseudomonas syringe* DC3000 and the non-pathogenic mutant strain *P. syringae* DC3000 (*hrcC^-^*). Bacterial growth was scored at 0 days post inoculation (dpi) and 3 dpi, respectively. Error bars indicate SD (*n* = 6). Letters above the bars indicate significant differences (*P* < 0.01). Similar results were obtained in three independent experiments.

XCT is a highly conserved protein across eukaryotes, and XAP5 domain at the C-terminus is the key functional domain. Therefore, truncation of this domain in *xct-5* mutation obviously affects its function. To examine how the *xct-5* mutation influences the function of *RPW8.1*, we measured some defense responses. First, we checked the distribution of cell death and production of H_2_O_2_ upon powdery mildew infection. Trypan blue staining of the inoculated leaf showed that clusters of dead cells were formed upon powdery mildew infection. The clusters of dead cells in R1Y4/*xct-5* were obviously less than those in R1Y4 at 10 dpi (**Figure 3A**). Consistently, DAB staining revealed that H_2_O_2_ accumulation in R1Y4/*xct-5* was less than that in R1Y4 (**Figure 3B**).

Then, we examined the defense responses induced by flg22. The results indicated that flg22-induced callose deposition was remarkably decreased in R1Y4/*xct-5* compared to that in R1Y4 (**Figure 3C**). We also examined the induced levels of defense-related genes, including *FLG22-INDUCED RECEPTOR-LIKE KINASE 1* (*FRK1, At2g19190*) and the *PATHOGENESIS - RELATED1* (*PR1, At2g19990*), by qRT-PCR. The level of *FRK1* was induced in R1Y4 upon application of flg22 and reached a summit at 6 h post application (hpa), then dropped back to relative lower levels at 12 and 24 hpa (**Figure 3D**). Similarly, the level of *FRK1* was also induced in R1Y4/*xct-5* upon application of flg22, but the amplitudes were significantly lower than those in R1Y4 at 3, 6, and 12 hpa (**Figure 3D**). The expression of *PR1* was induced and reached a summit at 24 hpa upon application of flg22 in both R1Y4 and R1Y4/*xct-5*, notably, its levels in R1Y4/*xct-5* were lower than those in R1Y4 at 0, 3, and 24 hpa (**Figure 3D**). These data indicate that *RPW8.1*-mediated defense responses were compromised by *xct-5* mutation.

Previously, we found that R1Y4 displayed enhanced resistance to the virulent bacterial strain *P. syringae* DC3000 and limited the proliferation of the non-pathogenic strain *P. syringae* DC3000 (*hrcC^-^*) (Li et al., 2017). We therefore tested the response of R1Y4/*xct-5* to these strains. Our data indicated that the proliferation of both *P. syringae* DC3000 and *P. syringae* DC3000 (*hrcC*^-^) in R1Y4/*xct-5* was significantly higher than that in R1Y4 and became comparable to that in Col-*gl* (**Figure 3E**). Together these data indicate that *XCT* is required for *RPW8.1*-mediated defense responses and resistance to different pathogens.

**FIGURE 4:**
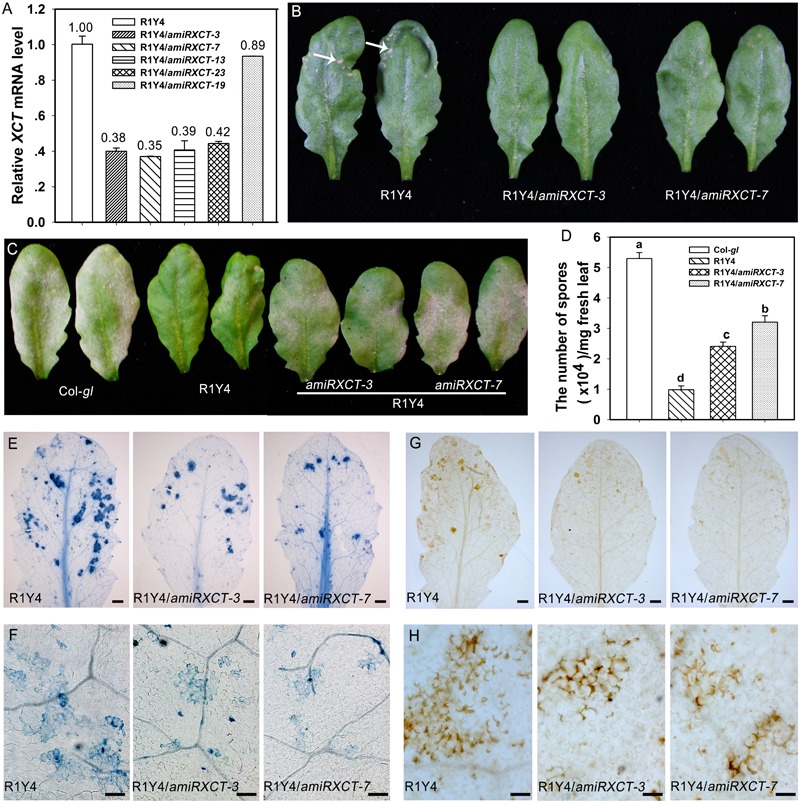
Knocking-down *XCT* compromises *RPW8.1*–mediated powdery mildew resistance. **(A)** Relative mRNA levels of *XCT* detected by quantitative RT-PCR (qRT-PCR) in the indicated transgene lines. The values above the column represent the relative level of *XCT* by arbitrarily setting the level of *XCT* in R1Y4 as 1. Error bars indicate SD (*n* = 3). **(B)** The phenotype of representative leaves from 6-week-old plants in R1Y4 compared to that in the *XCT* knocked-down lines. Note the cell death lesions in R1Y4 (arrows) but there are less or no such cell death lesions in the knocked-down lines. **(C)** Representative leaves show the disease phenotypes of powdery mildew at 10 dpi. **(D)** Quantitative analysis of sporulation at 10 dpi on indicated lines in **(C)**. Error bars represent SD (*n* = 6). Letters above the bars represent statistically significant differences (*P* < 0.01). **(E–H)** Representative leaves **(E,G)** and leaf sections **(F,H)** stained by trypan blue **(E,F)** and DAB **(G,H)** from R1Y4 and *XCT* knocked-down lines at 10 dpi show clusters of cell death and H_2_O_2_ accumulation, respectively. Scale bars: **(E,G)** 2 mm; **(F,H)** 50 μm.

**FIGURE 5:**
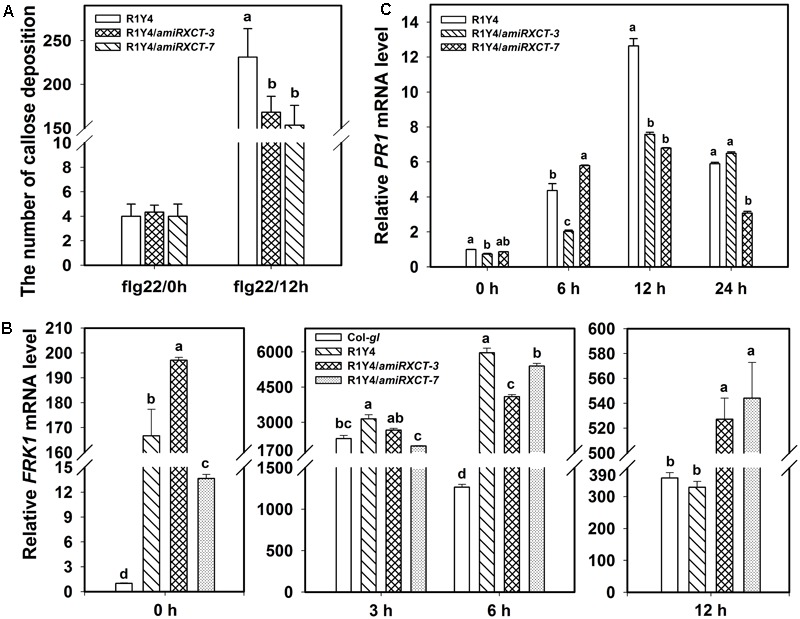
Knocking-down *XCT* compromises *RPW8.1*–mediated defense responses. **(A–C)** Quantification analysis on the number of callose deposition **(A)**, the relative level of *FRK1* **(B)** and *PR1* **(C)** induced by flg22 at the indicated time points in the indicated lines. Different letters above the bars indicate significant difference at *P* < 0.01. Similar results were obtained in three independent experiments.

To confirm that *xct-5* is a loss of function allele, we made a construct expressing an artificial microRNA that targets the 3′-UTR of *XCT* (*amiRXCT*, **Figure 2A**). The construct was introduced into R1Y4. Four lines in R1Y4 background exhibited remarkable reduction of *XCT* identified by qRT-PCR from 23 positive transformants (**Figure 4A**). The expression levels of *XCT* in the four lines were reduced to about 40% of that in R1Y4 (**Figure 4A**). Two of them, i.e., R1Y4/*amiRXCT-3* and R1Y4/*amiRXCT-7*, were selected for further experiments. The leaf size of R1Y4/*amiRXCT* lines was comparable with that of R1Y4, however, the cell death lesions were obviously less severe in R1Y4/*amiRXCT* lines than those of R1Y4 (**Figure 4B**). After inoculation with powdery mildew pathogen, the white fungus mass in the R1Y4/*amiRXCT* lines were obviously more than that in R1Y4, whereas, less than that in Col-*gl* (**Figure 4C**). Quantification analysis on spore numbers showed that the number of spores in R1Y4/*amiRXCT* lines was increased by about 2∼3 folds of that in R1Y4, but significantly lower than that in Col-*gl* (**Figure 4D**). These data indicate that the expression level of *XCT* is important for *RPW8.1*-mediated resistance to powdery mildew. Next, we examined the pathogen-induced cell death by trypan blue staining at 10 dpi of powdery mildew pathogen. The cell death lesion on the whole infected leaves in R1Y4/*amiRXCT* lines was obviously decreased compared to that in R1Y4 (**Figures 4E,F**). Consistently, the production of H_2_O_2_ revealed by DAB-staining was less in the R1Y4/*amiRXCT* lines than in R1Y4 (**Figures 4G,H**). We also examined flg22-induced callose deposition and the expression of *FRK1* and *PR1* in R1Y4/*amiRXCT* lines. The results indicated that callose deposited in R1Y4/*amiRXCT* lines was significantly less than those in R1Y4 (**Figure 5A**). Whereas, the basal level of *FRK1* in R1Y4 and the R1Y4/*amiRXCT* lines was significantly higher than that in Col-*gl*, and also significantly higher in one of the R1Y4/*amiRXCT* lines than that in R1Y4 at 0 hpa (**Figure 5B**). At 3 hpa, the level of *FRK1* became comparable in R1Y4/*amiRXCT* lines and R1Y4, but lower in R1Y4/*amiRXCT* lines than in R1Y4 at 6 hpa (**Figure 5B**). At 12 hpa, however, *FRK1* levels in the R1Y4/*amiRXCT* lines became higher than that in R1Y4 (**Figure 5B**), implying that in the R1Y4/*amiRXCT* lines the induction of *FRK1* by flg22 was delayed. Consistently, the level of *PR1* in R1Y4/*amiRXCT* lines was significantly lower than that in R1Y4 at 12 hpa, although in one of the R1Y4/*amiRXCT* lines was comparable with that in R1Y4 at 0, 6, and 24 hpa (**Figure 5C**). These results suggest that lower expression of *XCT* in R1Y4 compromised *RPW8.1*-mediated defense responses.

### Over-Expression of *XCT* Enhances *RPW8.1*-Mediated Cell Death and Disease Resistance

**FIGURE 6:**
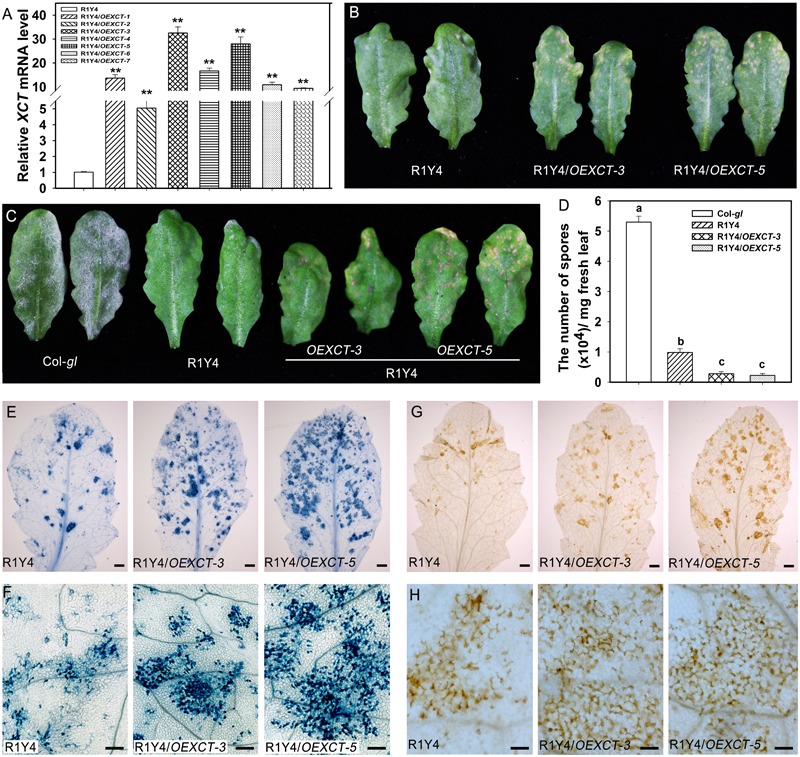
Overexpressing *XCT* enhances *RPW8.1*–mediated cell death and disease resistance. **(A)** Relative mRNA levels of *XCT* detected by quantitative RT-PCR (qRT-PCR) in the indicated transgene lines. Error bars indicate SD (*n* = 3). Asterisks ^∗∗^ above the bars indicate significant differences (*P* < 0.01) as determined by Student’s *t-*test between R1Y4 and the indicated transgenic lines (R1Y4/*XCTOE-1*∼R1Y4/*XCTOE-7*). **(B)** Representative leaves from the indicated lines of 5-week-old plants show the difference of cell death lesions. **(C)** Representative leaves show the disease phenotype of powdery mildew. Photos were taken at 10 dpi. **(D)** Quantitative analysis of sporulation at 10 dpi of the indicated lines. Error bars represent SD (*n* = 8). Letters above the bars indicate statistically significant differences at *P* < 0.01. **(E–H)** Representative leaves **(E,G)** and leaf sections **(F,H)** stained by trypan blue **(E,F)** and DAB **(G,H)** from R1Y4 and *XCT*-overexpressing lines at 10 dpi show clusters of cell death and H_2_O_2_ accumulation, respectively. Scale bars: **(E,G)** 2 mm; **(F,H)** 50 μm.

**FIGURE 7:**
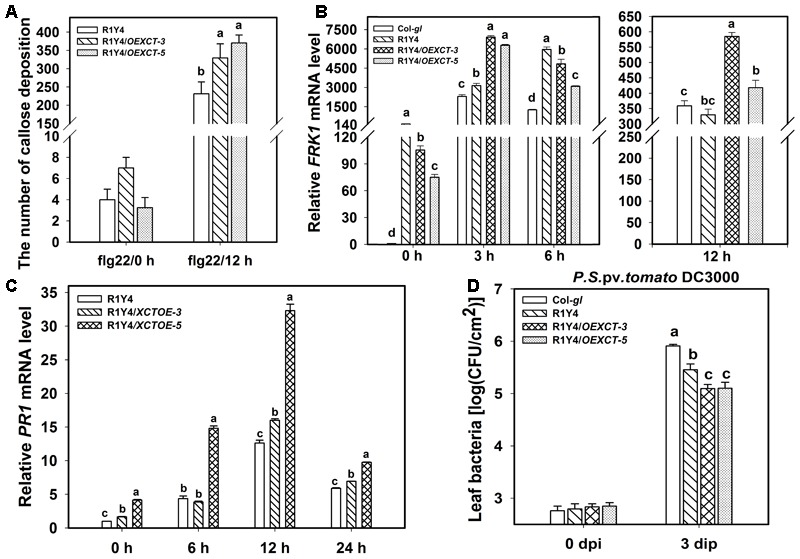
Overexpressing *XCT* enhances *RPW8.1*–mediated defense responses. **(A–C)** Quantification analysis on the number of callose deposition **(A)** and the relative level of *FRK1* **(B)** and *PR1* **(C)** induced by flg22 at the indicated time points in the indicated lines. Different letters above the bars indicate significant difference at *P* < 0.01. **(D)** Growth of *P. syringe* DC3000 at the indicated time points on the indicated lines. Error bars indicate SD (*n* = 6). Letters above the bars indicate significant differences (*P* < 0.01). Similar results were obtained in three independent experiments.

Now that mutation or down-regulation of *XCT* led to impairment of *RPW8.1*-mediated immunity, it was anticipated that over-expression of *XCT* should increase resistance. To this end, we made a construct overexpressing *XCT* from the constitutive 35S promoter (*OEXCT*). The construct was introduced into R1Y4 via Agrobacterium-mediated floral dip. More than 20 positive transformants were obtained and the expression level of *XCT* was examined by qRT-PCR (**Figure 6A**). Two lines (R1Y4/*OEXCT-3* and R1Y4/*OEXCT-5*) were selected for further experiments. The overexpression lines displayed enhanced cell death in comparison with R1Y4 in the absence of pathogen (**Figure 6B**), indicating than *XCT* positively regulated *RPW8.1*-mediated cell death. This observation prompted us to check if the mildew resistance in these lines was enhanced. Five-week-old plants were inoculated with powdery mildew and the disease phenotype was recorded at 10 dpi. While R1Y4 showed resistance in comparison with the noticeable susceptibility of Col-*gl*, there was hardly mycelium observed in R1Y4/*OEXCT-3* and R1Y4/*OEXCT-5* (**Figure 6C**). By quantitative analysis, the amount of spores in overexpression lines was significantly reduced than that in R1Y4 and Col-*gl*, respectively (**Figure 6D**). We also checked the spreading of cell death and accumulation of H_2_O_2_ in the overexpression lines. The clusters of dead cells in inoculated leaves of overexpression lines were obviously more than that of R1Y4 (**Figures 6E,F**). Similar to the cell death, overexpression lines displayed much more H_2_O_2_ production than R1Y4 (**Figures 6G,H**). Moreover, flg22-induced callose deposition was enhanced in R1Y4/*OEXCT-3* and R1Y4/*OEXCT-5* (**Figure 7A**). Flg-22 induced levels of *FRK1* were higher in R1Y4/*OEXCT-3* and R1Y4/*OEXCT-5* than that in R1Y4 at 3 hpa (**Figure 7B**). Flg-22 induced levels of *PR1* were also higher in R1Y4/*OEXCT* lines than that in R1Y4 (**Figure 7C**). Consistently, the proliferation of *Pst* DC3000 was reduced significantly in R1Y4/*OEXCT-3* and R1Y4/*OEXCT-5* in comparison with that in R1Y4 (**Figure 7D**). Taken together, these results demonstrated that up-regulation of *XCT* enhanced *RPW8.1*-mediated disease resistance and defense responses.

### *XCT* May Not Be Directly Involved in Defense

**FIGURE 8:**
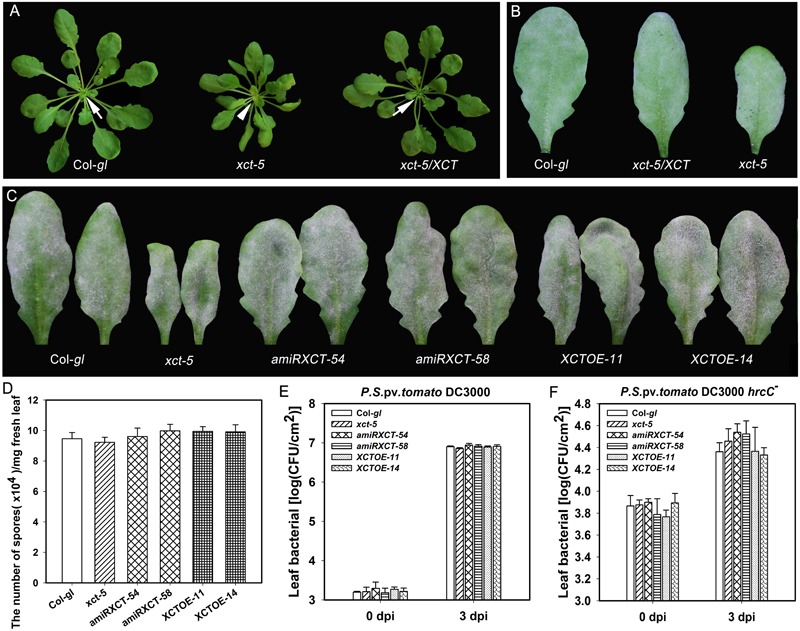
Mutation, knocking-down and overexpressing of *XCT* in Col-*gl* do not change disease phenotypes. **(A,B)** Representative plants **(A)** and powdery mildew infected-leaves **(B)** of the indicated lines show the morphology and disease phenotype, respectively. Note the freshly emerged light-green leaves in *xct-5* (arrowheads) in comparison with those in Col-*gl* and the complemented line *xct-5*/*XCT* (arrow). **(C)** Representative leaves from the indicated lines show the disease phenotype of powdery mildew at 10 dpi. **(D)** Quantification of sporulation at 10 dpi. Error bars represent SD (*n* = 6). **(E,F)** Growth of the virulent strain *P. syringae* DC3000 **(E)** and the non-pathogenic mutant strain *P. syringae* DC3000 (*hrcC^-^*) **(F)** in the indicated lines. Error bars indicate SD (*n* = 6). Similar results were obtained in three independent experiments.

Because mutation and down-regulation of *XCT* results in impairment, but overexpression of *XCT* leads to enhanced *RPW8.1*-mediated disease resistance, we asked whether *XCT* is directly involved in defense. To this end, we obtained *xct-5* mutant in Col-*gl* background by crossing *b3-17* to Col-*gl* and introduced the wild type *XCT* into *xct-5* to obtain the complemented lines with restored phenotypes (**Figure 8A**). Then we examined their responses to powdery mildew and two bacterial strains. Intriguingly, all these lines exhibited similar disease phenotypes of powdery mildew (**Figures 8B–D**). Furthermore, we also made transgenic lines expressing artificial microRNA or a *35S::XCT* construct in the Col-*gl* background, and surveyed for their defense responses. After examining the expression levels of *XCT* in these transgenic lines by qRT-PCR (**Supplementary Figure S2**), we selected two knocked-down lines (*amiRXCT-54* and *amiRXCT-58*) and two overexpression lines (*XCTOE-11* and *XCTOE-14*) for analysis on disease resistance. When inoculated with powdery mildew, the fungal mass in both knocked-down lines and overexpression lines was quite similar to that in Col-*gl*, indicating that these transgenic lines were as susceptible as Col-*gl* against powdery mildew (**Figures 8C,D**). Moreover, the proliferation of *Pst* DC3000 in *xct-5* mutant and the transgenic lines showed no difference from Col-*gl* (**Figure 8E**). In contrast, the proliferation of *Pst* DC3000 (*hrcC^-^*) was slightly higher in *xct-5* mutant and the knocked-down lines than that in Col-*gl* and the overexpression lines, but the differences were not significant (**Figure 8F**). These data demonstrate that *XCT* alone may not be directly involved in defense against powdery mildew and *P. syringae*.

### *XCT* and *RPW8.1* Are Mutually Regulated at Transcriptional Level

**FIGURE 9:**
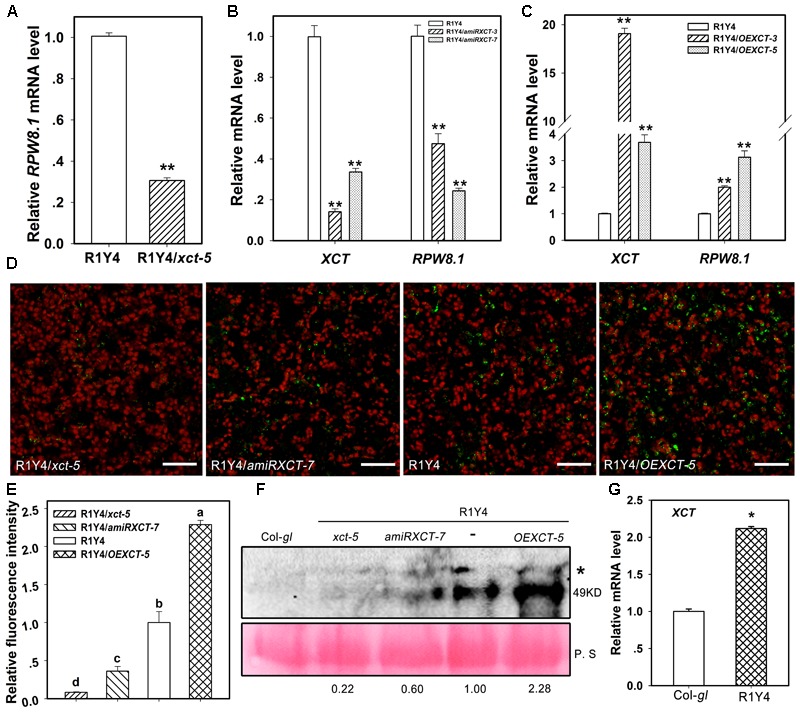
The expression of *RPW8.1* and *XCT* are mutually regulated. **(A–C)** Expression of *RPW8.1* and *XCT* from the indicated lines examined by qRT-PCR. Transcript levels were normalized to the internal control *ACT2*, and the levels in R1Y4 were arbitrarily set as 1.0. Error bars indicate SD (*n* = 3). Student’s *t-*test was performed to determine the significance between R1Y4 and the indicated lines. Asterisks ^∗∗^ indicate significant differences at P < 0.01. **(D)** Confocal images show the signal of RPW8.1-YFP fusion protein in the indicated lines at 5-week-old plants. The YFP fluorescence was pseudo-colored green and the auto-fluorescence of chloroplasts was pseudo-colored red. Scale bars, 20 μm. **(E)** Quantification analysis on the relative fluorescent intensity of YFP signal from **(D)**. The average value collected from more than 3 independent plants were processed by Image J. Error bars represent SD (*n* = 4). Letters above the bars indicate statistically significant differences (*P* < 0.05) by Tukey’s Honestly Significant Difference test. **(F)** Western blot analysis on the accumulation of RPW8.1-YFP in the indicated lines. RPW8.1-YFP was detected with GFP anti-sera that can also detect YFP. The band at molecular size of ∼49 kDa represents RPW8.1-YFP. A non-specific band was also detected (^∗^). Rubisco stained by Ponceau S was used as loading control. Values below the Rubisco indicate the relative abundance of RPW8.1-YFP by deducting the difference of loading and normalized to R1Y4 that was arbitrarily set as 1.0. Similar results were obtained in two independent experiments. **(G)** Relative mRNA level of *XCT* detected by quantitative RT-PCR from the indicated lines. Asterisk ^∗^ indicates significant differences at *P* < 0.01 (*t*-test). Similar results were obtained in three independent experiments.

It seems that *XCT* specifically and positively regulates *RPW8.1*-mediated resistance. From the cell death phenotype we speculated that the expression of *RPW8.1* may be altered by mutation or overexpression of *XCT*. To this end, we examined the mRNA level of *RPW8.1*. The transcription of *RPW8.1* in R1Y4*/xct-5* was reduced to about 30% of that in R1Y4 (**Figure 9A**). When the expression levels of *XCT* were down-regulated in R1Y4*/amiRXCT-3* and R1Y4*/amiRXCT-7* to about 20–40% of that in R1Y4, the levels of *RPW8.1* were decreased to ∼50% and 30% of that in R1Y4, respectively (**Figure 9B**). On the contrary, when the expression of *XCT* was increased in R1Y4/*OEXCT-3* and R1Y4/*OEXCT-5* by about 19- and 4-fold of that in R1Y4, the transcription of *RPW8.1* was increased to about 2- and 3-fold of that in R1Y4, respectively (**Figure 9C**). These results suggest that *XCT* positively regulate *RPW8.1* expression at transcriptional level.

To test if the accumulation of RPW8.1-YFP was consistent with its relative mRNA levels examined by qRT-PCR, we examined the fluorescent intensity of RPW8.1-YFP by Laser Scanning Confocal Microscopy (LSCM). The data showed that the RPW8.1-YFP signal was rarely found in leaves from R1Y4/*xct-5* and R1Y4/*amiRXCT-7* plants, whereas, the RPW8.1-YFP signal was obviously more in R1Y4/*OEXCT-5* than in R1Y4 (**Figure 9D**). Quantification analysis of the YFP signal intensity confirmed that the accumulation of RPW8.1-YFP in R1Y4/*xct-5* and R1Y4/*amiRXCT-7* was significantly lower than that in R1Y4, whereas the accumulation of RPW8.1-YFP in the overexpression line was significantly higher than that in R1Y4 (**Figure 9E**). We next checked the expression of RPW8.1-YFP by western blotting analysis using GFP polyclonal antibody that could also detect YFP. We found that RPW8.1-YFP expression was higher in OE lines by ∼2 folds compared to that in R1Y4, whereas the expression of RPW8.1-YFP was barely detected in R1Y4/*xct-5* and reduced in R1Y4/*amiRXCT-*7 (**Figure 9F**). These results suggest that *XCT* positively regulate RPW8.1 expression.

Now that *XCT* can regulate the transcription of *RPW8.1*, we asked whether *RPW8.1* can also affect the expression of *XCT*. To this end, we compared the expression of *XCT* in R1Y4 and Col-*gl*. Intriguingly, the expression level of *XCT* in R1Y4 was increased to ∼2-fold of that in Col-*gl* (**Figure 9G**), indicating that the expression of *RPW8.1* can also up-regulate the expression of *XCT*.

Therefore, it appears that *XCT* and *RPW8.1* can mutually enhance each other’s transcription.

## Discussion

*RPW8.1* is among the few broad-spectrum resistance genes characterized. Previously, we found that ectopic expression of RPW8.1-YFP can boost PTI to enhance resistance against different pathogens in Arabidopsis and rice (Ma et al., 2014; Li et al., 2017). Full function of *RPW8.1*-mediated resistance to powdery mildew requires proper expression of *AS1* (Zhao et al., 2015). Here, we demonstrated that *XCT* positively regulates *RPW8.1*-mediated cell death and disease resistance. In a forward genetic screen, we identified the *b3-17* mutant that exhibited susceptibility to powdery mildew (**Figure 1**). Map-based cloning identified that the *b3-17* mutant contained a novel allele of *XCT* (**Figure 2** and **Supplementary Figure S1**). Both mutation and down-regulation of *XCT* led to impairment of *RPW8.1*-mediated defense responses (**Figures 3–5**). On the contrary, over-expression of *XCT* resulted in enhanced *RPW8.1*-mediated cell death and resistance to pathogens (**Figures 6, 7**). Therefore, *XCT* acts as a positive regulator for the *RPW8.1*-mediated defense pathway.

How *XCT* regulates *RPW8.1*-mediated defense is an intriguing question. Previous reports may give some clues to explain why *XCT* can regulate *RPW8.1*-mediated defense. *XCT* is initially identified in a genetic screen for circadian clock mutants (Martin-Tryon and Harmer, 2008). Loss-of-function of *XCT* leads to pleiotropic phenotypes, such as short-period circadian rhythms, delayed greening, altered regulation of hypocotyl elongation, constitutively enhanced ethylene responses, and compromised small RNA production (Martin-Tryon and Harmer, 2008; Ellison et al., 2011; Fang et al., 2015). Therefore, *XCT* may affect the function of *RPW8.1* and regulate the expression of *RPW8.1* through several aspects.

*XCT* may act as a transcriptional regulator through manipulation of chromatin properties to regulate directly or indirectly the expression of certain genes such as *RPW8.1*. In *Schizosaccharomyces pombe*, XAP5 is a chromatin-associated protein localized at both the genic and intergenic regions to suppress the expression of antisense and repeat elements, and the yeast *xap5* mutant can be completely rescued by the Arabidopsis *XCT* (Anver et al., 2014), indicating conserved roles of *XCT* in manipulation of chromatin properties. The nuclear localization feature of XCT implies its roles in the nucleus (Martin-Tryon and Harmer, 2008). A transcriptional regulator role of *XCT* can also explain a previous observation that the occupancy of Pol II at *DCL1, DCL3*, and *DCL4* is decreased in the *xct-2* mutant leading to the reduced expression of these genes, which in turn results in the decreased production of small RNAs (Fang et al., 2015). Additionally, proper chromatin maintenance is known to be important for normal plant growth and development (Van Driel et al., 2003). Our data show that the expression of *RPW8.1* was down- and up-regulated in mutant and overexpression lines, respectively (**Figure 9**). The protein level of RPW8.1-YFP was lesser abundant in R1Y4/*xct-5* than in R1Y4 (**Figure 9F**). Therefore, *XCT* plays a role to regulate the expression of *RPW8.1*. However, whether *XCT* directly or indirectly regulates the expression of *RPW8.1* is unclear, and this could be a good research focus in the future.

Defect of chloroplast pigment caused by *xct* mutation may reduce RPW8.1-triggered ROS production in chloroplast. Chloroplasts play important roles in production and transportation of defense-related signal molecules, such as ROS and salicylic acid signals during immune responses (Caplan et al., 2015; De et al., 2015). We observed the delayed greening phenotype of *xct-5* plants in both R1Y4 and Col-*gl* background (**Figures 1, 2, 8**). Given that RPW8.1 is associated with chloroplasts in its localization and triggers ROS/H_2_O_2_ production in chloroplasts (Li et al., 2017), normal chloroplast pigment may be required for RPW8.1-triggered production of ROS/H_2_O_2_ in chloroplast, which in turn leads to cell death. Conversely, over-expression of *XCT* may facilitate *RPW8.1*-triggered production of ROS/H_2_O_2_ so as to promote cell death (**Figure 6**).

Alternatively, *XCT* may regulate the expression of *RPW8.1* through the ethylene-signaling pathway. *XCT* negatively regulates the ethylene-signaling pathway down-stream of *EIN3* and loss-of *XCT* leads to constitutively enhanced ethylene responses (Ellison et al., 2011). Previously, *AS1* is found to be required for *RPW8.1*-mediated resistance to powdery mildew (Zhao et al., 2015). In fact, *AS1* also negatively regulates the ethylene-signaling pathway in addition to its function in development (Nurmberg et al., 2007). The nucleus-localized protein XCT was proposed to affect the stability of a subset of *ERF* genes downstream of *EIN3* (Ellison et al., 2011). In addition, *ERF6* was reported to act as a negative regulator of the ROS signaling (Sewelam et al., 2013). Therefore, there could be possible connection between *RPW8.1, AS1, XCT* and the ethylene-signaling. Because both *XCT* and *AS1* are mutually up-regulated with *RPW8.1* (Figure 9, Zhao et al., 2015), *XCT* and *AS1* could act together in suppression of the ethylene-signaling pathway to promote *RPW8.1*’s expression. Although the exact mechanism is currently unknown, we propose a working hypothesis that the ethylene-signaling may negatively regulate the expression of *RPW8.1* and proper expression of *RPW8.1* would require the suppression of the ethylene-signaling by *XCT* together with *AS1*. However, experimental evidence is currently lacking and this could be another good research focus in the future.

Moreover, the short-period rhythm in *xct* mutant may down-regulate the expression of *RPW8.1*. Circadian clock functions in multiple biological processes and is important for plant health. Recently, growing evidence indicates that circadian clock also plays a critical role in plant immunity, and both short-period mutants and arrhythmic plants exhibit higher susceptibility to pathogens (Lu et al., 2017). This is consistent with the case that *xct* is a short-period mutant (Martin-Tryon and Harmer, 2008). In Arabidopsis, Col-0 displays a fluctuant response to the virulent bacterial pathogen *Pst* DC3000, showing resistance in the morning but susceptibility in the evening (Bhardwaj et al., 2011). Moreover, a large number of defense-related genes show a circadian-regulation model, including genes encoding the flagellin receptor and proteins in the MKK4/5-MPK3/6-WRKY22 signal cascade (Bhardwaj et al., 2011). Another study showed that disrupting the core components of circadian clock (*CCA1* and *LHY*), which play a synergistically role in controlling clock activity, led to more severe susceptibility to bacterial and oomycete pathogens (Zhang et al., 2013). Data gathered from the above reports reveal an important crosstalk between circadian rhythm and innate immunity in plants. In the present study, *XCT* was identified as a positive regulator of *RPW8.1*-mediated resistance against powdery mildew, but itself does not seem to contribute to defense. It is possible that *RPW8.1*-mediated resistance is linked to the circadian rhythm via the expression of *XCT*. However, how *XCT* and *RPW8.1* mutually regulates expression at transcriptional level is another interesting question for future investigation.

Whether there are any microRNA genes involved in regulation of *RPW8.1*-mediated defense is another open question. Increasing evidence indicates microRNAs are important regulators of plant innate immunity (Weiberg et al., 2014). Because the production of microRNAs is decreased in the *xct* mutant (Fang et al., 2015), it is anticipated that the function of certain microRNAs could be associated with *RPW8.1*-mediated defense. It is intriguing that loss-of-function and over-expression of *XCT* did not show significant impact on defense in Col-*gl* background (**Figure 8**), however, they may generate some alteration in defense responses at certain time point of a day that we did not detect. In fact, we observed marginal difference in the growth of the non-pathogenic strain *P. syringae* DC3000 (*hrcC^-^*) in the *xct-5* mutant and the down-regulated lines (**Figure 8F**). Such alteration could become more significant upon expression of *RPW8.1*, which could result in the compromised resistance to pathogens in R1Y4/*xct-5* and enhanced cell death in R1Y4/*OEXCT* (**Figures 4–7**).

Taken together, *XCT* is a pleiotropic gene with several separable functions in plant growth and immunity. Our results demonstrate that *XCT* contributes to *RPW8.1* expression and *RPW8.1*-mediated resistance against pathogens. However, the exact molecular mechanism underlying the connection between *XCT* and *RPW8.1* is yet unknown. Future works should be focused on investigation of the potential mechanism by which *XCT* positively regulates *RPW8.1* expression.

## Author Contributions

Y-JX, YL, RL, L-LZ, Z-XZ, J-HZ, HY, and JS performed the experiments. SX, YL, JF, and W-MW supervised the study. Y-JX and W-MW wrote the manuscript. SX and W-MW coordinated the overall study and edited the manuscript.

## Conflict of Interest Statement

The authors declare that the research was conducted in the absence of any commercial or financial relationships that could be construed as a potential conflict of interest.

## Footnotes

1 http://wmd3.weigelworld.org

## Supplementary Material

FIGURE S1 Map-based cloning of *b3-17.* **(A)** Schematic graph shows map-based cloning of the *b3-17* mutant gene. A horizontal black bar represents chromosome 2. Markers were shown above the bar and their physical locations on the chromosome were shown by numbers below the bar. BAC, bacterial artificial chromosome. **(B)** Schematic gene structure of *XCT* (*At2g21150*). The exons and introns in the coding region of the candidate gene were shown as black boxes and gray bars, respectively. **(C)** Alignment of the cDNA of *XCT* from *b3-17* with wild type *XCT* cDNA and genome DNA shows the altered splice in the mutant (red box). **(D)** Alignment of the putative amino acid (AA) residues between b3-17 and the wild type XCT (AT2G21150) shows the induction of stop site after two AA substitutions (red ^∗^in the red box).

FIGURE S2 Quantitative analyses on the relative mRNA level of *XCT.* **(A,B)** Relative mRNA level examined by quantitative RT-PCR in the representative knocked-down **(A)** and overexpression lines **(B)**. Error bars indicate SD (*n* = 3). Student’s *t*-test was carried out to determine the significance of difference between Col-*gl* and the indicated transgenic lines. Asterisk ^∗^ and ^∗∗^ indicated significant difference at *P* ≤ 0.05 and *P* ≤ 0.01, respectively. Similar results were obtained in two experiments.

## References

[B1] Anver S. Roguev A. Zofall M. Krogan N. J. Grewal S. I. S. Harmer S. L. (2014). Yeast X-chromosome-associated protein 5 (Xap5) functions with H2A.Z to suppress aberrant transcripts. *EMBO Rep.* 15 894–902. 10.15252/embr.201438902 24957674 PMC4197047

[B2] Asai T. Tena G. Plotnikova J. Willmann M. R. Chiu W. L. Gomezgomez L. (2002). MAP kinase signalling cascade in *Arabidopsis* innate immunity. *Nature* 415 977–983. 10.1038/415977a 11875555

[B3] Bhardwaj V. Meier S. Petersen L. N. Ingle R. A. Roden L. C. (2011). Defence responses of *Arabidopsis thaliana* to infection by *Pseudomonas syringae* are regulated by the circadian clock. *PLOS ONE* 6:e26968. 10.1371/journal.pone.0026968 22066021 PMC3205005

[B4] Boller T. Felix G. (2009). A renaissance of elicitors: perception of microbe-associated molecular patterns and danger signals by pattern-recognition receptors. *Annu. Rev. Plant Biol.* 60 379–406. 10.1146/annurev.arplant.57.032905.105346 19400727

[B5] Bonardi V. Cherkis K. Nishimura M. T. Dangl J. L. (2012). A new eye on NLR proteins: focused on clarity or diffused by complexity? *Curr. Opin. Immunol.* 24 41–50. 10.1016/j.coi.2011.12.006 22305607 PMC3482489

[B6] Brueggeman R. Rostoks N. Kudrna D. Kilian A. Han F. Chen J. (2002). The barley stem rust-resistance gene *Rpg1* is a novel disease-resistance gene with homology to receptor kinases. *Proc. Natl. Acad. Sci. U.S.A.* 99 9328–9333. 10.1073/pnas.142284999 12077318 PMC123140

[B7] Caplan J. L. Kumar A. S. Park E. Padmanabhan M. S. Hoban K. Modla S. (2015). Chloroplast stromules function during innate immunity. *Dev. Cell.* 34 45–57. 10.1016/j.devcel.2015.05.011 26120031 PMC4596411

[B8] Clough S. J. Bent A. F. (1998). Floral dip: a simplified method for *Agrobacterium*-mediated transformation of *Arabidopsis thaliana*. *Plant J.* 16 735–743. 10.1046/j.1365-313x.1998.00343.x 10069079

[B9] Dangl J. L. Horvath D. M. Staskawicz B. J. (2013). Pivoting the plant immune system from dissection to deployment. *Science* 341 746–751. 10.1126/science.1236011 23950531 PMC3869199

[B10] Dangl J. L. Jones J. D. (2001). Plant pathogens and integrated defence responses to infection. *Nature* 411 826–833. 10.1038/35081161 11459065

[B11] De T. Z. M. Littlejohn G. Jayaraman S. Studholme D. Bailey T. Lawson T. (2015). Chloroplasts play a central role in plant defence and are targeted by pathogen effectors. *Nat. Plants* 1:15074. 10.1038/nplants.2015.74 27250009

[B12] Dodds P. N. Lawrence G. J. Catanzariti A. M. Teh T. Wang C. A. Ayliffe M. A. (2006). Direct protein interaction underlies gene-for-gene specificity and coevolution of the flax resistance genes and flax rust avirulence genes. *Proc. Natl. Acad. Sci. U.S.A.* 103 8888–8893. 10.1073/pnas.0602577103 16731621 PMC1482673

[B13] Dou D. Zhou J. M. (2012). Phytopathogen effectors subverting host immunity: different foes, similar battleground. *Cell Host Microbe* 12 484–495. 10.1016/j.chom.2012.09.003 23084917

[B14] Ebel J. Mithöfer A. (1998). Early events in the elicitation of plant defence. *Planta* 206 335–348. 10.1007/s004250050409

[B15] Ellison C. T. Vandenbussche F. Straeten D. V. D. Harmer S. L. (2011). *XAP5 CIRCADIAN TIMEKEEPER* regulates ethylene responses in aerial tissues of Arabidopsis. *Plant Physiol.* 155 988–999. 10.1104/pp.110.164277 21163961 PMC3032482

[B16] Fang X. Shi Y. Lu X. Chen Z. Qi Y. (2015). CMA33/XCT regulates small RNA production through modulating the transcription of *Dicer-Like* genes in *Arabidopsis*. *Mol. Plant* 8 1227–1236. 10.1016/j.molp.2015.03.002 25770820

[B17] Greenberg J. T. Yao N. (2004). The role and regulation of programmed cell death in plant-pathogen interactions. *Antiquity* 85 729–741. 10.1111/j.1462-5822.2004.00361.x 14764104

[B18] Huang Y. Y. Shi Y. Lei Y. Li Y. Fan J. Xu Y. J. (2014). Functional identification of multiple nucleocytoplasmic trafficking signals in the broad-spectrum resistance protein RPW8.2. *Planta* 239 455–468. 10.1007/s00425-013-1994-x 24218059

[B19] Jones J. D. Dangl J. L. (2006). The plant immune system. *Nature* 444 323–329. 10.1038/nature05286 17108957

[B20] Krasileva K. V. Dahlbeck D. Staskawicz B. J. (2010). Activation of an *Arabidopsis* resistance protein is specified by the in planta association of its leucine-rich repeat domain with the cognate oomycete effector. *Plant Cell* 22 2444–2458. 10.1105/tpc.110.075358 20601497 PMC2929106

[B21] Krattinger S. G. Lagudah E. S. Spielmeyer W. Singh R. P. Huerta-Espino J. McFadden H. (2009). A putative ABC transporter confers durable resistance to multiple fungal pathogens in wheat. *Science* 323 1360–1363. 10.1126/science.1166453 19229000

[B22] Li Y. Zhang Y. Wang Q. X. Wang T. T. Cao X. L. Zhao Z. X. (2017). *RESISTANCE TO POWDERY MILDEW8.*1 boosts pattern-triggered immunity against multiple pathogens in Arabidopsis and rice. *Plant Biotechnol. J.* 10.1111/pbi.12782 [Epub ahead of print]. 28640974 PMC5787827

[B23] Livak K. J. Schmittgen T. D. (2001). Analysis of relative gene expression data using real-time quantitative PCR and the 2^-ΔΔC_T_^ method. *Methods Mol. Biol.* 25 402–408. 10.1006/meth.2001.1262 11846609

[B24] Lu H. McClung C. R. Zhang C. (2017). Tick Tock: circadian regulation of plant innate immunity. *Annu. Rev. Phytopathol.* 55 287–311. 10.1146/annurev-phyto-080516-35451 28590878

[B25] Ma X. F. Li Y. Sun J. L. Wang T. T. Fan J. Lei Y. (2014). Ectopic expression of *RESISTANCE TO POWDERY MILDEW8.1* confers resistance to fungal and oomycete pathogens in Arabidopsis. *Plant Cell Physiol.* 55 1484–1496. 10.1093/pcp/pcu080 24899552

[B26] Martin G. B. Brommonschenkel S. H. Chunwongse J. Frary A. Ganal M. W. Spivey R. (1993). Map-based cloning of a protein kinase gene conferring disease resistance in tomato. *Science* 262 1432–1436. 10.1126/science.7902614 7902614

[B27] Martin-Tryon E. L. Harmer S. L. (2008). *XAP5 CIRCADIAN TIMEKEEPER* coordinates light signals for proper timing of photomorphogenesis and the circadian clock in *Arabidopsis*. *Plant Cell* 20 1244–1259. 10.1105/tpc.107.056655 18515502 PMC2438460

[B28] Nurmberg P. L. Knox K. A. Yun B. W. Morris P. C. Shafiei R. Hudson A. (2007). The developmental selector AS1 is an evolutionarily conserved regulator of the plant immune response. *Proc. Natl. Acad. Sci. U.S.A.* 104 18795–18800. 10.1073/pnas.0705586104 18003921 PMC2141856

[B29] Schwab R. Ossowski S. Riester M. Warthmann N. Weigel D. (2006). Highly specific gene silencing by artificial microRNAs in *Arabidopsis*. *Plant Cell* 18 1121–1133. 10.1105/tpc.105.039834 16531494 PMC1456875

[B30] Sewelam N. Kazan K. Thomas-Hall S. R. Kidd B. N. Manners J. M. Schenk P. M. (2013). Ethylene response factor 6 is a regulator of reactive oxygen species signaling in *Arabidopsis*. *PLOS ONE* 8:e70289. 10.1371/journal.pone.0070289 23940555 PMC3734174

[B31] Spoel S. H. Dong X. (2012). How do plants achieve immunity? Defence without specialized immune cells. *Nat. Rev. Immunol.* 12 89–100. 10.1038/nri3141 22273771

[B32] Van Driel R. Fransz P. F. Verschure P. J. (2003). The eukaryotic genome: a system regulated at different hierarchical levels. *J. Cell Sci.* 116 4067–4075. 10.1242/jcs.00779 12972500

[B33] Wang W. Devoto A. Turner J. G. Xiao S. (2007). Expression of the membrane-associated resistance protein RPW8 enhances basal defense against biotrophic pathogens. *Mol. Plant Microbe Interact.* 20 966–976. 10.1094/MPMI-20-8-0966 17722700

[B34] Wang W. Wen Y. Berkey R. Xiao S. (2009). Specific targeting of the *Arabidopsis* resistance protein RPW8.2 to the interfacial membrane encasing the fungal Haustorium renders broad-spectrum resistance to powdery mildew. *Plant Cell* 21 2898–2913. 10.1105/tpc.109.067587 19749153 PMC2768920

[B35] Wang W. Zhang Y. Wen Y. Berkey R. Ma X. Pan Z. (2013). A comprehensive mutational analysis of the *Arabidopsis* resistance protein RPW8.2 reveals key amino acids for defense activation and protein targeting. *Plant Cell* 25 4242–4261. 10.1105/tpc.113.117226 24151293 PMC3877822

[B36] Wang W. M. Ma X. F. Zhang Y. Luo M. C. Wang G. L. Bellizzi M. (2012). PAPP2C interacts with the atypical disease resistance protein RPW8.2 and negatively regulates salicylic acid-dependent defense responses in *Arabidopsis*. *Mol. Plant* 5 1125–1137. 10.1093/mp/sss008 22334594

[B37] Weiberg A. Wang M. Bellinger M. Jin H. (2014). Small RNAs: a new paradigm in plant-microbe interactions. *Annu. Rev. Phytopathol.* 52 495–516. 10.1146/annurev-phyto-102313-145933 25090478

[B38] Xiao S. Brown S. Patrick E. Brearley C. Turner J. G. (2003). Enhanced transcription of the Arabidopsis disease resistance genes *RPW8.1* and *RPW8.2* via a salicylic acid-dependent amplification circuit is required for hypersensitive cell death. *Plant Cell* 15 33–45. 10.1105/tpc.006940 12509520 PMC143449

[B39] Xiao S. Ellwood S. Calis O. Patrick E. Li T. Coleman M. (2001). Broad-spectrum mildew resistance in *Arabidopsis thaliana* mediated by *RPW8*. *Science* 291 118–120. 10.1126/science.291.5501.118 11141561

[B40] Zhang C. Xie Q. Anderson R. G. Ng G. Seitz N. C. Peterson T. (2013). Crosstalk between the circadian clock and innate immunity in Arabidopsis. *PLOS Pathog.* 9 344–351. 10.1371/journal.ppat.1003370 23754942 PMC3675028

[B41] Zhao Z. X. Xu Y. B. Wang T. T. Ma X. F. Zhao J. Q. Li Y. (2015). Proper expression of AS1 is required for RPW8.1-mediated defense against powdery mildew in Arabidopsis. *Physiol. Mol. Plant Pathol.* 92 101–111. 10.1016/j.pmpp.2015.09.002

[B42] Zipfel C. Kunze G. Chinchilla D. Caniard A. Jones J. D. G. Boller T. (2006). Perception of the bacterial PAMP EF-Tu by the receptor EFR restricts *Agrobacterium* -mediated transformation. *Cell* 125 749–760. 10.1016/j.cell.2006.03.037 16713565

[B43] Zipfel C. Robatzek S. Navarro L. Oakeley E. J. Jones J. D. Felix G. (2004). Bacterial disease resistance in *Arabidopsis* through flagellin perception. *Nature* 428 764–767. 10.1038/nature02485 15085136

