# Supplementary figures and images for: XAP5 CIRCADIAN TIMEKEEPER Positively Regulates RESISTANCE TO POWDERY MILDEW8.1–Mediated Immunity in Arabidopsis
Source: Front Plant Sci. 2017 Nov 30;8:2044. doi: 10.3389/fpls.2017.02044 (PMC5714888; doi:10.3389/fpls.2017.02044)

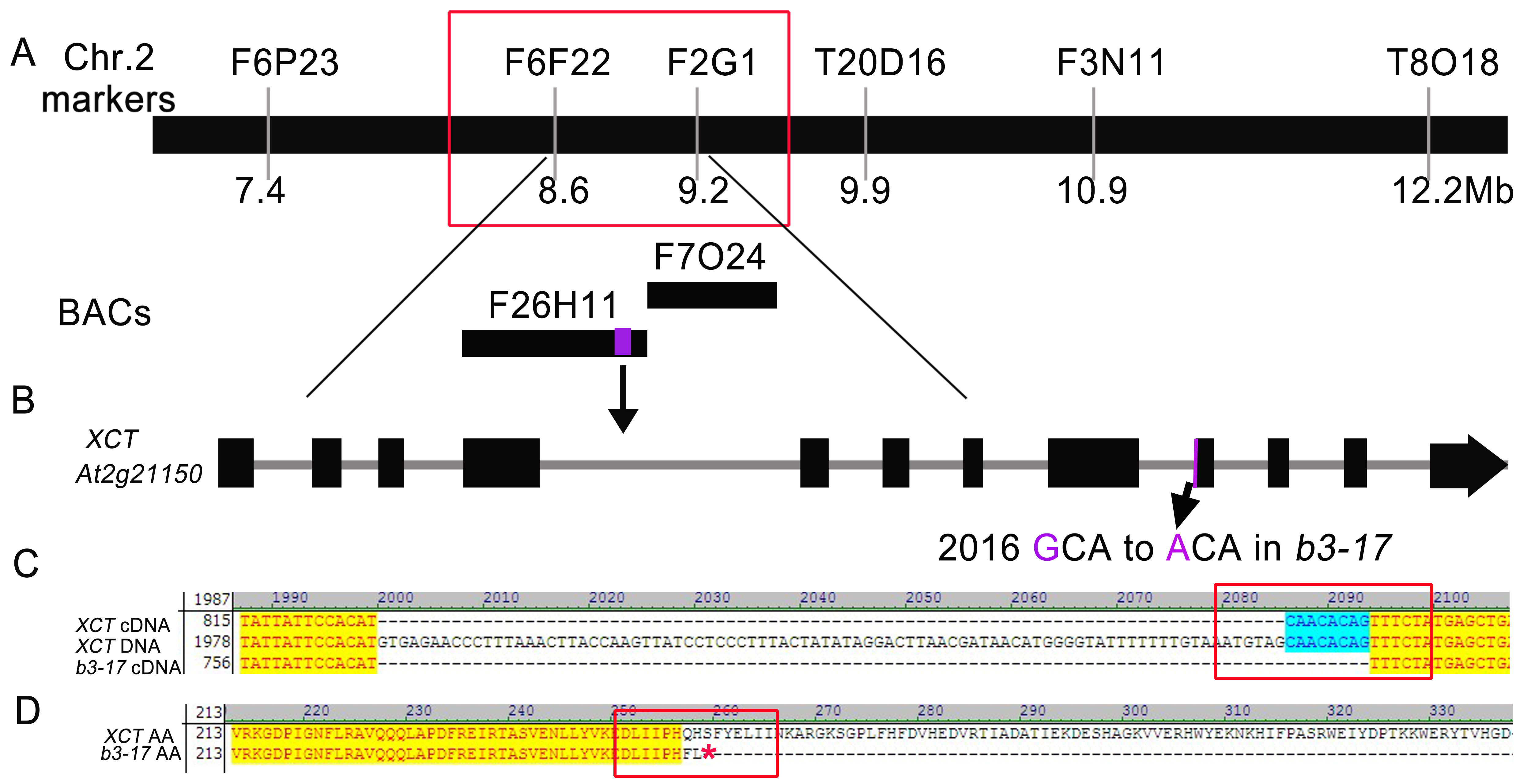

Supplement: FIGURE S1 — Map-based cloning of b3-17. (A) Schematic graph shows map-based cloning of the b3-17 mutant gene. A horizontal black bar represents chromosome 2. Markers were shown above the bar and their physical locations on the chromosome were shown by numbers below the bar. BAC, bacterial artificial chromosome. (B) Schematic gene structure of XCT (At2g21150). The exons and introns in the coding region of the candidate gene were shown as black boxes and gray bars, respectively. (C) Alignment of the cDNA of XCT from b3-17 with wild type XCT cDNA and genome DNA shows the altered splice in the mutant (red box). (D) Alignment of the putative amino acid (AA) residues between b3-17 and the wild type XCT (AT2G21150) shows the induction of stop site after two AA substitutions (red ∗in the red box). [file Image_1.JPEG]

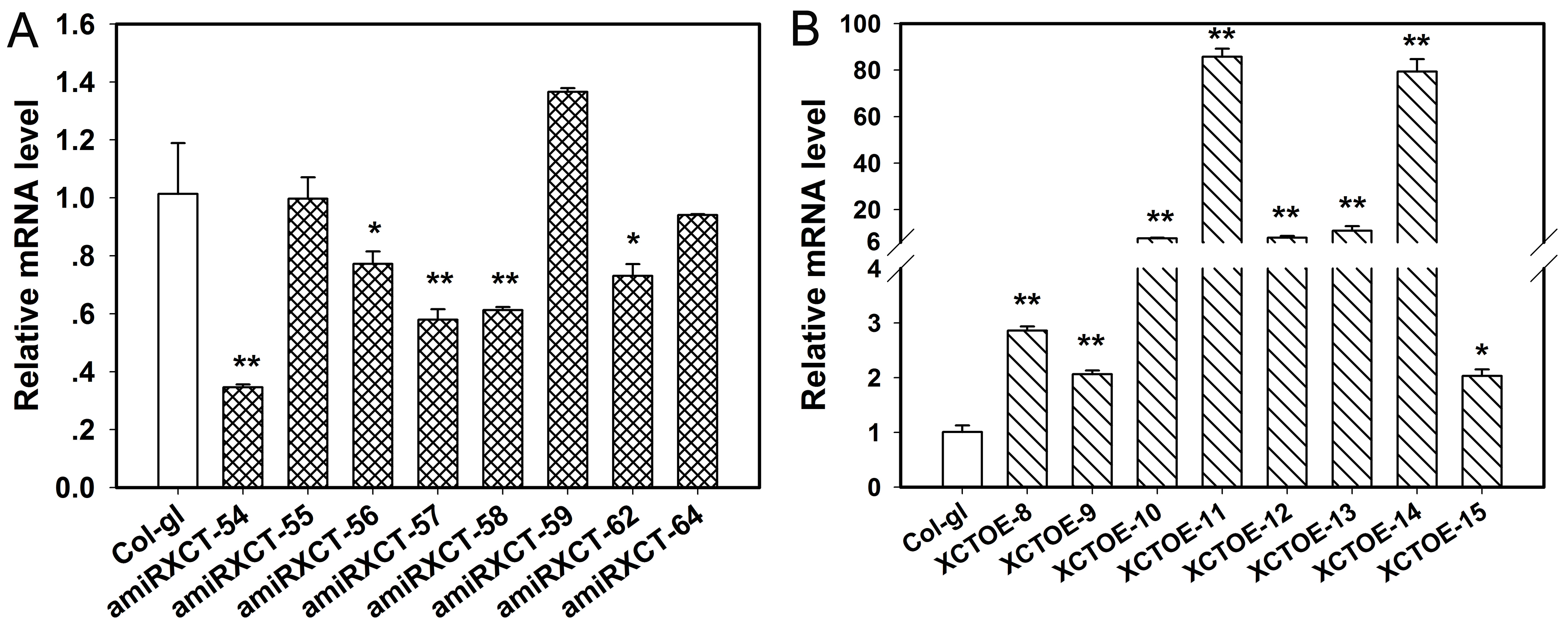

Supplement: FIGURE S2 — Quantitative analyses on the relative mRNA level of XCT. (A,B) Relative mRNA level examined by quantitative RT-PCR in the representative knocked-down (A) and overexpression lines (B). Error bars indicate SD (n = 3). Student’s t-test was carried out to determine the significance of difference between Col-gl and the indicated transgenic lines. Asterisk ∗ and ∗∗ indicated significant difference at P ≤ 0.05 and P ≤ 0.01, respectively. Similar results were obtained in two experiments. [file Image_2.JPEG]
